# Does increased body mass index lead to elevated prostate cancer risk? It depends on waist circumference

**DOI:** 10.1186/s12885-020-07089-5

**Published:** 2020-06-23

**Authors:** Jin Bong Choi, Jun-Pyo Myong, Yunhee Lee, Inah Kim, Jung Ho Kim, Sung-Hoo Hong, U-Syn Ha

**Affiliations:** 1 Department of Urology, Bucheon St. Mary’s Hospital, College of Medicine, The Catholic University of Korea, Seoul, South Korea; 2 Department of Occupational and Environmental Medicine, Seoul St. Mary’s Hospital, College of Medicine, The Catholic University of Korea, Seoul, South Korea; 3 Department of Occupational and Environmental Medicine, Hanyang University College of Medicine, Seoul, South Korea; 4 Department of Urology, Dongnam Institute of Radiological & Medical Sciences, Cancer Center, Busan, South Korea; 5 Department of Urology, Seoul St. Mary’s Hospital, College of Medicine, The Catholic University of Korea, 222, Banpo-daero, Seocho-gu, Seoul, 06591 South Korea

**Keywords:** Prostate cancer, Obesity, Waist circumference

## Abstract

**Background:**

We examined the association between obesity and prostate cancer based on both body mass index (BMI) and waist circumference (WC) using the National Health Insurance System (NHIS) database for the entire male population of Korea.

**Methods:**

A total of 1,917,430 men who underwent at least one health examination in 2009 without a previous diagnosis of any other cancer were tracked through December 2015. The hazard ratio (HR) and 95% confidence interval (CI) value for the association between prostate cancer and obesity were analyzed using multiple Cox regression model. Since there was a statistically significant interaction between WC and BMI, a multiple HR for prostate cancer was estimated with stratifying both WC and BMI to control the interaction between WC and BMI.

**Results:**

Without considering WC as an adjustment factor, very weak association between BMI and prostate cancer development risk was observed. When WC was considered as an adjustment factor, no significant change in the HRs for prostate cancer development beyond the reference BMI was observed in the group with WC < 85 cm in the multivariable-adjusted models. However, in the group with WC ≥ 85 cm, the HRs for prostate cancer increased as the BMI increased beyond the reference BMI. In addition, there was a discrepancy in the trend of prostate cancer development according to BMI among the groups with different categories for WC.

**Conclusion:**

In groups with abdominal obesity, a significant linear relationship was observed between increasing BMI and prostate cancer risk. Higher the WC category, the stronger was the association with BMI, signifying that the association of BMI with risk of prostate cancer development depends on abdominal obesity.

## Background

Prostate cancer is the second most common cancer in men worldwide and the third most commonly occurring cancer in both the sex according to GLOBOCAN 2018 [1, 2]. Though African-American men have still the highest incidence rate, a constant increase has been reported in many Asian countries [3]. In Korea, the age-standardized rate for prostate cancer incidence has increased by 0.2% annually from 1999 to 2016 [4]. Therefore, prostate cancer can be considered to one of the most important male cancers in Korea.

This increasing trend may partly be explained by rapid population aging, westernized dietary habits, and increased prostate-specific antigen (PSA) screening [5]. In addition to these risk factors, obesity, which showed an upward trend among men, has been reported as a high-risk factor of prostate cancer in Korea [6]. Associations between obesity and prostate cancer risk have also been analyzed in multiple studies worldwide [7–10]. However, these studies have varied substantially in their results depending on the research methods used.

The existing discrepancies might be because most of the studies used body mass index (BMI) to analyze the relationship between obesity and prostate cancer. Though BMI provides the most common estimate of obesity in cancer epidemiologic studies, it does not measure adipose mass sufficiently [11]. Especially in men, BMI correlates better with lean mass than with body adiposity, and it is hypothesized that metabolically obese but normal-weight (MONW) individuals might have a normal BMI [12].

Consequently, other measures that could capture centralized fat disposition such as waist circumference (WC) should be estimated in combination with BMI when considering obesity as a risk factor for prostate cancer development. Therefore, in this study, we examined the association between obesity and prostate cancer based on both BMI and WC using the National Health Insurance System (NHIS) database for the entire male population of Korea. 

## Methods

### Data source

The NHIS database of Korea is a public database comprising of the eligibility database, the national health screening database, the health care utilization database, and the long-term care insurance database [13]. This database includes the insurance claim code based on the International Classification of Diseases, 10th Revision, Clinical Modification (ICD-10-CM). Prostate cancer is coded C61. To identify clearly the diagnosis of prostate cancer, patients who had not undergone trans-rectal prostate biopsy were excluded from the study. In addition, the national health screening database was used to obtain information about WC, BMI, and other variables (hypertension, diabetes, dyslipidemia, alcohol consumption status, and smoking status).

The definition of measurements including categories for WC and BMI has been described in previous study using the NHIS database as follows: 1) WC cutoff value for abdominal obesity: ≥ 90 cm for men, 2) BMI categories: underweight (under 18.5), normal weight (18.5 to 22.9), overweight (23 to 24.9), obese class 1 (25 to 29.9), obese class 2 (over 30), 3) Hypertension: diagnostic code I10–15, blood pressure ≥ 140/90 mmHg, or history of taking antihypertensive drugs, 4) Diabetes: diagnostic code E10–14, fasting serum glucose level ≥ 126 mg/dl, or self-reported medical history of diabetic drugs, and 5) Dyslipidemia: diagnostic code E78, total cholesterol level ≥ 240 mg/dl, or self-reported use of lipid-lowering drugs [14].

### Study population

**Fig. 1:**
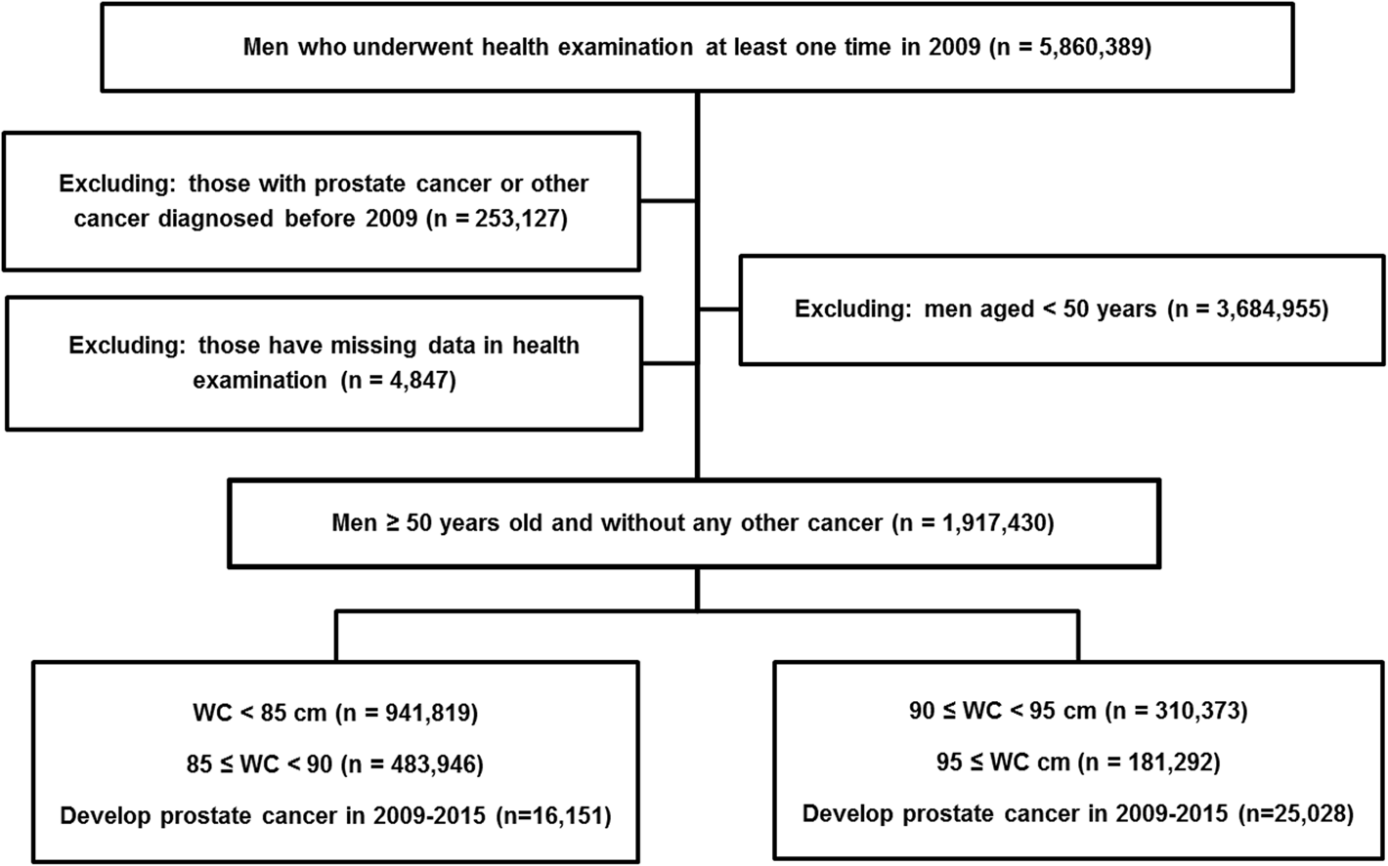
Study design and disposition of subjects

Of the 5,860,389 men who underwent at least one health examination in 2009, men with prostate cancer or other cancers diagnosed before 1^st^ January 2009 (*n* = 253,157) were excluded. Moreover, patients aged < 50 years (*n* = 3,684,955) were excluded because prostate cancer is rare in this age group. After excluding people with missing WC or BMI data from health examination databases (*n* = 4847), a total of 1,917,430 men without a previous diagnosis of any other cancer were tracked through 31^th^ December 2015. The study design and disposition of the subjects are shown in Fig. 1.

### Statistical analysis

The baseline characteristics of subjects have been presented as the number (%) for categorical variables. Incidence rate is expressed as the number of newly diagnosed cases of prostate cancer per 100,000 person-years of the follow-up period. The hazard ratio (HR) and 95% confidence interval (CI) value for prostate cancer of obesity were analyzed using a multiple Cox proportional hazard model. The model was adjusted for common variables such as age, diabetes, hypertension, dyslipidemia, alcohol consumption, and smoking status; the obesity indices (BMI and WC) were alternatively added on common variables for adjustment in multiple Cox proportional hazard model. There was a statistically significant interaction between WC and BMI (*P* value < 0.05). To control the interaction between WC and BMI on the incidence of prostate cancer, a multiple HR for prostate cancer was estimated with stratifying both obesity indices (WC; four classes and BMI; five classes) were classified into, respectively. Statistical significance for linear trends in the HRs for prostate cancer of BMI and WC were verified at a level of 0.05. SAS software (version 9.4, SAS Institute, Cary, NC, USA) was used for statistical analyses.

## Results

### Baseline clinical characteristics of study population according to categories for WC

Among a total of 1,917,430 men, 22,584 (1.18%) incident cases of prostate cancer developed between the beginning of 2009 and the end of 2015 (Table 1). Approximately 1.31% of the population categorized as WC ≥ 90 cm was diagnosed with prostate cancer, while 1.13% of the population categorized as WC < 90 cm was diagnosed with prostate cancer. In the obesity group categorized as WC ≥ 90 cm, 3.05% had a normal weight according to the BMI, and 20.65% of the men categorized as the non-obesity group (WC < 90 cm) were obese class 1 according to the BMI. In addition, the obesity group was more likely to have hypertension (54.45% vs. 36.25%), dyslipidemia (26.72% vs. 18.14%), and diabetes (20.65% vs. 13.65%).

**Table 1:** Clinical characteristics of population according to WC

Abdominal obesity			Non-obesity group (WC < 90)			Obesity group (90 ≤ WC)	
WC, cm	WC < 85	85 ≤ WC < 90		90 ≤ WC < 95	WC ≥ 95		Total
No. in population	941,819	483,946	1,425,765	310,373	181,292	491,665	1,917,430
No. of diagnosed prostate cancer	10,273 (1.09)	5878 (1.21)	16,151 (1.13)	3989 (1.29)	2444 (1.35)	6433 (1.31)	22,584 (1.18)
Age ≥ 65	266,825 (28.33)	127,838 (26.42)	394,663 (27.68)	88,811 (28.61)	57,504 (31.72)	146,315 (29.76)	540,978 (28.21)
BMI, kg/m^2^							
< 18.5	45,695 (4.85)	622 (0.13)	46,317 (3.25)	0	0	0	46,317 (2.42)
18.5–22.9	543,779 (57.74)	79,125 (16.35)	622,904 (43.69)	12,949 (4.17)	2054 (1.13)	15,003 (3.05)	637,907 (33.27)
23.0–24.9	262,548 (27.88)	199,528 (41.23)	462,076 (32.41)	71,087 (22.9)	11,493 (6.34)	82,580 (16.79)	544,656 (28.41)
25.0–29.9	89,797 (9.53)	204,671 (42.29)	294,468 (20.65)	220,303 (70.98)	132,657 (73.17)	352,960 (71.79)	647,428 (33.77)
≥ 30	0	0	0	6034 (1.94)	35,088 (19.35)	41,122 (8.36)	41,122 (2.14)
Smoking status							
Non	318,875 (34.04)	166,136 (34.53)	485,011 (34.01)	108,223 (35.06)	64,466 (35.77)	172,689 (35.12)	657,700 (34.49)
Former	264,566 (28.24)	160,015 (33.26)	424,581 (29.77)	105,633 (34.23)	62,080 (34.45)	167,713 (34.11)	592,294 (31.06)
Current	353,311 (37.72)	155,024 (32.22)	508,335 (35.65)	94,785 (30.71)	53,658 (29.78)	148,443 (30.19)	656,778 (34.44)
Alcohol consumption	521,926 (58.02)	278,409 (60.33)	800,335 (56.13)	177,492 (60.04)	101,238 (58.7)	278,730 (56.69)	1,079,065 (58.99)
Hypertension	306,479 (32.54)	210,423 (43.48)	516,902 (36.25)	158,373 (51.03)	109,334 (60.31)	267,707 (54.45)	784,609 (40.92)
Dyslipidemia	150,954 (16.03)	107,752 (22.27)	258,706 (18.14)	78,911 (25.42)	52,463 (28.94)	131,374 (26.72)	390,080 (20.34)
Diabetes	116,357 (12.35)	78,387 (16.2)	194,744 (13.65)	58,963 (19)	42,600 (23.5)	101,563 (20.65)	296,307 (15.45)

Data are presented as the number (%)

*BMI* body mass index, *WC* waist circumference

### Risk of prostate cancer according to BMI or WC

The HR for prostate cancer was lowest in subjects with a BMI <  18.5 and highest in those with 23.0 ≤ BMI < 25 in the multivariate-adjusted model (HR 0.93, 95% CI 0.85 to 1.02 and HR 1.12, 95% CI 1.08 to 1.16). However, the HRs for prostate cancer showed a decreasing trend with BMI over 25. Even in the patients with BMI ≥ 30, the 95% CI reflected the relative risk of 1.00. So, when we did not consider WC as an adjustment factor, there was a very weak association between BMI and prostate cancer development risk. The risk of prostate cancer according to WC also showed no statistically significant difference among the groups without stratification by BMI (Table 2). We also found that there was a statistically significant interaction between WC and BMI in this cohort (*P* value < 0.05).

**Table 2:** Multivariate-adjusted HRs for prostate cancer according to BMI and WC

	Event	Person-years	Incidence^a^	HR (95% confidence interval) ^b^	*P* value
BMI, kg/m^2^					
< 18.5	466	322,751	144.38	0.93 (0.85, 1.02)	0.143
18.5–22.9	7338	4,841,077	151.57	Ref.	
23.0–24.9	6775	4,218,668	160.59	1.12 (1.08, 1.16)	< .001
25.0–29.9	7560	5,039,457	150.01	1.05 (1.03, 1.08)	0.007
≥ 30	445	319,345	139.34	0.98 (0.89, 1.08)	0.650
WC, cm					
< 85	3833	7,206,600	142.54	0.91 (0.88, 0.94)	< .001
85–90	4260	3,744,368	156.98	Ref.	
90–95	4363	2,398,016	166.34	0.99 (0.95, 1.03)	0.703
≥ 95	716	1,392,314	175.53	0.97 (0.92, 1.02)	0.179
^c^*P* for interaction	< 0.05				

*BMI* body mass index, *WC* waist circumference, *HR* hazard ratio

^a^All rates are expressed as number per 100,000 person-years

^b^Adjusted for age, diabetes, hypertension, dyslipidemia, smoking status, and alcohol consumption

^c^*P* for interaction between BMI and WC

### The association between BMI and prostate cancer development according to WC

**Fig. 2:**
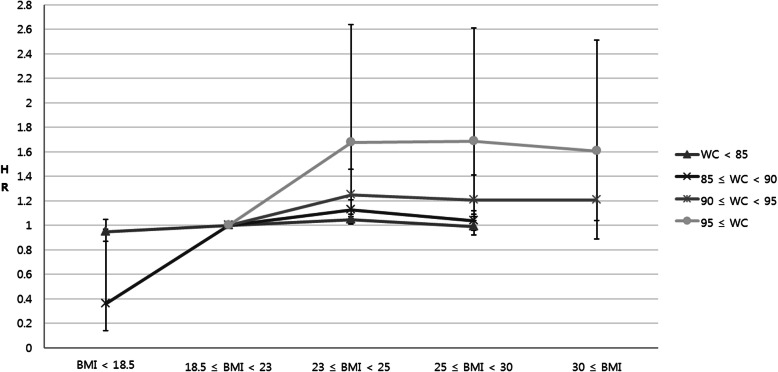
Multivariable-adjusted hazard ratios for prostate cancer according to BMI stratified by WC. The error bars represent the upper and lower limits of the 95% confidence interval

Since there was a statistically significant interaction between WC and BMI, a multiple HR for prostate cancer was estimated with stratifying both WC and BMI to control the interaction between WC and BMI. In the group with WC < 85 cm, there was no significant change in the HRs for prostate cancer development beyond the reference BMI in the multivariable-adjusted model (*P* for trend = 0.158; Table 3 and Fig. 2). The HR (95% CI) was 1.05 (1.01, 1.09) in the overweight group, which was characterized by a BMI over 23, and 0.99 (0.92, 1.07) in class 1 obese group, which was characterized by a BMI over 25.

**Table 3:** Age- and multivariable-adjusted HRs for prostate cancer according to BMI stratified by WC

					HR (95% confidence interval)	
WC, cm	BMI, kg/m^2^	Event	Person-years	Incidence^a^	Model 1^b^	Model 2^c^
WC < 85	< 18.5	462	318,733	144.95	1.06 (0.96, 1.16)	0.95 (0.87, 1.05)
	18.5–22.9	6033	4,138,307	145.78	Ref.	Ref.
	23.0–24.9	2874	2,046,401	140.44	0.97 (0.93, 1.02)	1.05 (1.01, 1.09)
	25.0–29.9	904	703,159	128.56	0.89 (0.83, 0.96)	0.99 (0.92, 1.07)
	≥ 30	0	0	0	–	–
85 ≤ WC < 90	< 18.5	4	4018	99.55	0.50 (0.19, 1.34)	0.36 (0.14, 0.97)
	18.5–22.9	1086	594,059	182.81	Ref.	Ref.
	23.0–24.9	2638	1,544,705	170.78	0.95 (0.88, 1.02)	1.13 (1.04, 1.21)
	25.0–29.9	2150	1,601,586	134.24	0.76 (0.70, 0.81)	1.04 (0.96, 1.12)
	≥ 30	0	0	0	–	–
90 ≤ WC < 95	< 18.5	0	0	0	–	–
	18.5–22.9	196	94,269	207.91	Ref.	Ref.
	23.0–24.9	1065	542,265	196.39	0.97 (0.83, 1.13)	1.25 (1.06, 1.46)
	25.0–29.9	2665	1,714,191	155.47	0.79 (0.68, 0.91)	1.21 (1.04, 1.41)
	≥ 30	63	47,291	133.22	0.68 (0.52, 0.90)	1.21 (0.89, 1.62)
95 ≤ WC	< 18.5	0	0	0	–	–
	18.5–22.9	23	14,442	159.26	Ref.	Ref.
	23.0–24.9	198	85,297	232.13	1.47 (0.96, 2.27)	1.68 (1.07, 2.64)
	25.0–29.9	1841	1,020,521	180.39	1.19 (0.79, 1.80)	1.69 (1.09, 2.61)
	≥ 30	382	272,054	140.41	0.95 (0.63, 1.45)	1.61 (1.04, 2.51)

*BMI* body mass index, *WC* waist circumference, *HR* hazard ratio

^a^All rates are expressed as number per 100,000 person-years

^b^Adjusted for age

^c^Adjusted for age, diabetes, hypertension, dyslipidemia, smoking status, and alcohol consumption

However, in the groups with WC ≥ 85 cm (groups with 85 ≤ WC < 90, 90 ≤ WC < 95, and WC ≥ 95), the HRs for prostate cancer increased as the BMI increased beyond the reference BMI (*P* for trend < .001). In addition, there was a discrepancy in the trend of prostate cancer development according to BMI among the groups with different categories for WC (Fig. 2). The increasing rate of prostate cancer development risk according to BMI was greatest in the group with WC ≥ 95. On the other hand, HRs for prostate cancer according to WC stratified by BMI showed that impact of BMI on the risk of prostate cancer according to WC was weak (Table 4).

**Table 4:** Age- and multivariable-adjusted HRs for prostate cancer according to WC stratified by BMI

					HR (95% confidence interval)	
BMI, kg/m^2^	WC, cm	Event	Person-years	Incidence^a^	Model 1^b^	Model 2^c^
< 18.5	WC < 85	462	318,733	144.95	Ref.	Ref.
	85 ≤ WC < 90	4	4018	99.55	0.47 (0.18, 1.26)	0.48 (0.18, 1.27)
	90 ≤ WC < 95	0	0	0	–	–
	95 ≤ WC	0	0	0	–	–
18.5–22.9	WC < 85	6033	4,138,307	145.75	Ref.	Ref.
	85 ≤ WC < 90	1086	594,059	182.81	1.16 (1.09, 1.24)	1.12 (1.05, 1.20)
	90 ≤ WC < 95	196	94,269	207.91	1.14 (0.99, 1.31)	1.03 (0.89, 1.20)
	95 ≤ WC	23	14,442	159.26	0.81 (0.54, 1.22)	0.72 (0.47, 1.11)
23.0–24.9	WC < 85	2874	2,046,401	140.44	Ref.	Ref.
	85 ≤ WC < 90	2638	1,544,705	170.78	1.08 (1.03, 1.15)	1.09 (1.03, 1.15)
	90 ≤ WC < 95	1065	542,265	196.39	1.04 (0.97, 1.12)	1.05 (0.98, 1.13)
	95 ≤ WC	198	85,297	232.13	0.99 (0.86, 1.15)	0.98 (0.85, 1.14)
25.0–29.9	WC < 85	904	703,159	128.56	Ref.	Ref.
	85 ≤ WC < 90	2150	1,601,586	134.24	1.01 (0.93, 1.09)	1.01 (0.93, 1.09)
	90 ≤ WC < 95	2665	1,714,191	155.27	1.04 (0.96, 1.12)	1.03 (0.95, 1.11)
	95 ≤ WC	1841	1,020,521	180.39	1.03 (0.95, 1.11)	1.01 (0.93, 1.09)
≥ 30	WC < 85	0	0	0	–	–
	85 ≤ WC < 90	0	0	0	–	–
	90 ≤ WC < 95	63	47,291	133.22	Ref.	Ref.
	95 ≤ WC	382	272,054	140.41	0.93 (0.71, 1.21)	0.92 (0.69, 1.21)

*BMI* body mass index, *WC* waist circumference, *HR* hazard ratio

^a^All rates are expressed as number per 100,000 person-years

^b^Adjusted for age

^c^Adjusted for age, diabetes, hypertension, dyslipidemia, smoking status, and alcohol consumption

**Fig. 3:**
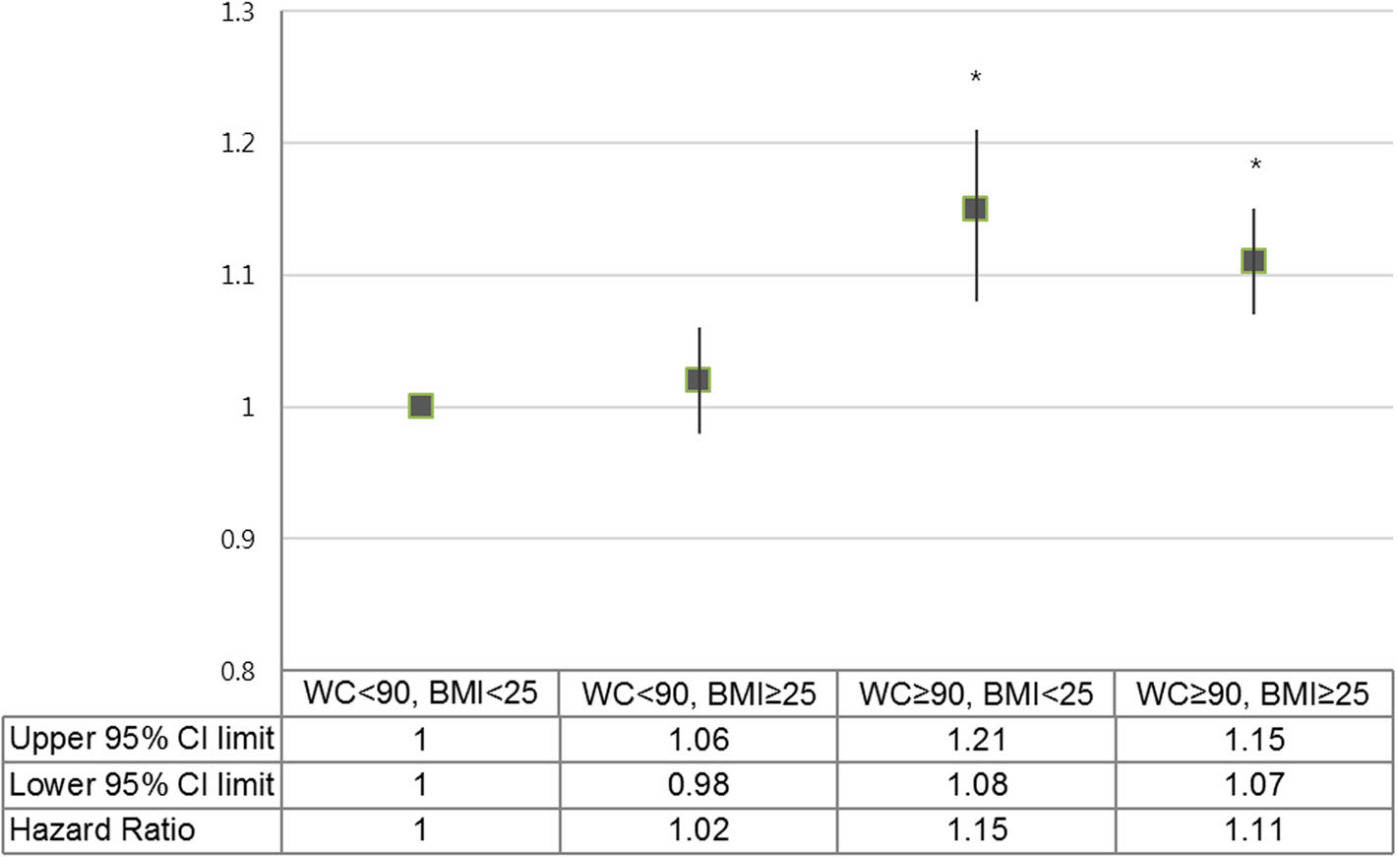
Hazard ratio for prostate cancer development according to BMI (comparison of BMI < 25 and BMI ≥ 25) when stratified by abdominal obesity (comparison of WC < 90 and WC ≥ 90) in the age-adjusted model. The error bars represent the upper and lower limits of the 95% confidence interval. ^*^P value < .001

Figure 3 shows the HRs for prostate cancer development according to BMI (comparison of BMI < 25 and BMI ≥ 25) when stratified based on abdominal obesity (comparison of WC < 90 and WC ≥ 90). The HR for prostate cancer was significantly higher in WC ≥ 90 groups regardless of BMI than the reference group (*P* value < .001). However, in the WC < 90 groups, HR did not show a significant difference even if BMI was higher than 25 (*P* value = 0.301).

## Discussion

The main findings of this population-based study are (1) without considering WC as an adjustment factor, very weak association between BMI and prostate cancer development risk was observed; (2) when WC was considered as an adjustment factor, there were significant linear relations between increasing BMI and prostate cancer risk in the groups with abdominal obesity; (3) the higher the WC category, the stronger association with BMI was noted; and (4) this means that the association of BMI with risk of prostate cancer development depends on abdominal obesity.

Although the terms “overweight” and “obese” are similar, the difference between the two depends on the BMI. Although BMI is the most widely used method of measuring obesity, an imperfect measurement does not accurately reflect adiposity. BMI indicates overweight relative to height, but it does not discriminate between fat mass and lean body mass. Body composition is variable among individuals with the same BMI [11, 12]. Therefore, majority of the reported studies based on BMI provide an agreement that obesity is a risk factor for aggressive prostate cancer development, whereas other studies provide contrary results about localized or overall prostate cancer risk [15–17]. The inconsistent findings might be due to limitations in BMI as a calculation of adiposity.

In the present study, low WC and high BMI may be indicative of high lean body mass. In the low WC group (WC < 85 cm), adiposity of men with high BMI is expected to be smaller due to high lean body mass. Furthermore, there was a discrepancy in the trend of prostate cancer development according to BMI among the groups with different categories for WC. This finding signifies that in the higher WC group, men with high BMI have more adiposity than in the lower WC group, which increases the risk of prostate cancer development.

The distinctive feature of our study is that though few studies have simultaneously evaluated the associations between BMI, WC, and prostate cancer risk in the west [18, 19], to the best of our knowledge, this is the first study to evaluate the risk for patients in an Asian population with prostate cancer stratified by BMI and WC. Especially, it is noteworthy that our results are based on the Asian population. Due to ethnic differences in body composition like muscle mass and fat distribution between Westerners and Asians [20, 21], research on evaluating risk of prostate cancer development based on obesity in Asians is necessitated.

Because obesity also affects cancer screening in various aspects, research on the relationship between obesity and cancer development is important. Increasing BMI is associated with a decrease in serum PSA, which may minimize the diagnosis of prostate cancer based on PSA screening. When considering indications for prostatic biopsy in obese men, we should be aware that the effect of hemodilution might reduce PSA levels [22, 23]. Obesity has been correlated with large prostate volume [24]. In case of larger prostate volume, it becomes difficult to get a diagnosis of prostate cancer in the same number of samples. Additionally, with adiposity impeding physical examinations, obese men may face difficulties in undergoing a DRE due to body habitus hindering prostate access [25]. Chu et al. reported that the predictive value of DRE is dependent on obesity and is significantly higher among obese men than normal-weight men [26]. Because DRE may be limited by adiposity in obese men, cancers that are large enough to be palpable may be more meaningful. Consequently, the clinician should remind these effects during the diagnostic process in patients with high BMI or high WC.

The major limitation of our study is that detailed information, such as digital rectal examination (DRE) finding, serum PSA level, prostate volume, cancer stage, Gleason grade, and molecular pathology, were not available for this nationwide cohort; thus, we could not adjust for these factors. The treatment patterns and the overall prognosis differed among regionally localized intermediate, high, and very high-risk prostate cancer. In addition, as previous studies have reported different effects of obesity [15–17], failure to perform stage analysis is an important limitation of our study. Therefore, detailed information should be thoroughly considered in future etiologic research.

## Conclusions

Without considering WC as an adjustment factor, very weak association between BMI and prostate cancer development risk was observed. However, when we considered WC as an adjustment factor, in the groups with abdominal obesity, significant linear relationship was observed between increasing BMI and prostate cancer risk, and the higher the WC category, the stronger association with BMI was noted. This means that the association of BMI with risk of prostate cancer development depends on abdominal obesity. Therefore, WC should be mutually estimated while considering obesity as a risk factor of prostate cancer development.

## Data Availability

The data supporting the findings of this study are available from National Health Insurance Sharing Service (https://nhiss.nhis.or.kr) in Korea, but are not publicly available due to restrictions on the availability of the materials used under the permission of this study.

## Abbreviations

BMI: Body mass index
WC: Waist circumference
NHIS: National Health Insurance System
PSA: Prostate-specific antigen
DRE: Digital rectal examination
